# Thermal Stability of Chromium-Iron Oxidation Mixture Cermet-Based Solar Selective Absorbing Coatings

**DOI:** 10.3390/molecules25051178

**Published:** 2020-03-05

**Authors:** Hongwen Yu, Jinkai Li, Qian Zhang, Wei Pang, Hui Yan, Guangyuan Li

**Affiliations:** 1 College of Materials Science and Engineering, Key Laboratory of Advanced Functional Materials of Education Ministry of China, Beijing Key Laboratory of Microstructure and Property of Advanced Materials, Beijing University of Technology, Beijing 100124, China; hongwen_yu@163.com (H.Y.); 13646399507@163.com (Q.Z.); pw724976911@163.com (W.P.); 2 School of Materials Science and Engineering, University of Jinan, Jinan 250022, China; mse_lijk@ujn.edu.cn; 3 Energy Research Institute, Qilu University of Technology (Shandong Academy of Sciences), Jinan 250014, China

**Keywords:** chemical coloring, thermal shock, chromium-iron oxidation mixture cermet, solar selective absorber coating

## Abstract

The solar selective absorber coating (SSAC) are at the core of the efficient solar-thermal system. In this paper, for the first time, the Chromium-iron oxidation mixture cermet was successfully prepared on the surface of ultra-pure ferritic stainless steel by chemical coloring as SSAC. The coating surface has an optical trap structure, and the chromium-iron oxidation mixture cermet is used as an absorption layer to realize solar-thermal conversion. The solar absorptance (AM1.5) of the coating reached 93.66, and the thermal emittance was less than 13. After thermal shock tests at 25/300 °C done 32 times (accumulated 812.8 h), the Performance Criterion (PC) of the coating was 0.01375 < 0.05, showing outstanding thermal stability.

## 1. Introduction

Under the current trend of global energy shortage, the energy structure of the world is undergoing significant changes [1,2]. According to the analysis of global energy consumption in 2017, industrial energy accounted for 32% of global energy consumption, and 74% of industrial energy belongs to industrial heat. In total, 52% of industrial heat belongs to (below 400 °C) medium–low-term and can be provided by solar or supplementary energy [3]. A variety of industrial processes demand vast amounts of thermal energy, which makes the industrial sector a promising market for solar thermal applications [4]. The SSAC with excellent performance is the core to realize the high efficiency thermal conversion of solar energy [5]. The ideal SSAC requires high solar absorptance (α) in 300-2500 nm and low emittance (ε) [6,7]. With the continuous expansion of solar-thermal applications, the working temperature of solar collectors continues to increase [8,9], and the thermal stability requirements of coatings at high temperatures are constantly increasing. Some transition metal nitrides, oxides and nitrogen oxides, possessing with excellent thermal stability at high temperatures [10], the forbidden band width of ideal, good solar absorptance and thermal emittance, are gradually becoming a research hot spot.

Traditional solar-thermal substrate is mainly copper, aluminum etc. [11], using PVD and CVD technology in preparation of substrate [12,13], the solar absorptance of the coating is generally more than 0.9 and emittance is less than 0.2 [14]. In the future, the solar-thermal will be an important expansion direction at 120–250 °C [15]. Restricting the application of coatings to medium and high temperatures is the heat resistance of the coating. Another key issue is structural defects of the substrate with exposed to high and low temperature conversion for a long time [14,16]. At present, stainless steel is used to prepare medium- and high-temperature coating substrate [17,18]. Researchers generally use PVD to prepare coating [6,18]. This method has the advantages of accurate control of various control parameters and good optical properties, but the disadvantage is that the bonding strength between coating and stainless steel substrate is low [19,20], and the thermal shock resistance is poor, which affects the marketing promotion. 

Chemical coloring is a process for the preparation of color stainless steel [21]. A variety of metal ceramic functional coatings, such as corrosion resistance, wear resistance, heat resistance, oxidation resistance and so on [22], can be prepared on the surface of stainless steel by adding current. With low cost, easy operation, high bonding strength between coating and substrate (generally up more than to 40 MPa) [23], good heat shock resistance, the coating can be produced in large scale and mass production. However, the preparation of SSAC by chemical coloring has only some patents of Nippon Steel, Mitsubishi and other companies [24], and there is no detailed report on the effect of preparation process on the spectral properties of the coatings.

The service life of solar-thermal system is generally required to reach 20–30 years, so the thermal stability of SSAC is a very important indicator. Many studies have been conducted on the thermal stability of coatings [8,25]. Standard EN12975.3-1 specifies the Performance Criterion (PC) of the coating after several rounds of heat treatment experiments. If the PC value of the coating is no more than 0.05, this indicates that the coating can achieve the expected service life of 25 a [14]. PC is calculated using Equation (1), where Δα = α(aged) – α(unaged), Δε = ε(aged) – ε(unaged), PC are taking the absolute value.

(1) PC=Δα−0.5Δε

However, under actual working conditions, the SSAC of solar energy is faced with the alternating day and night cycle thermal shock, so how to effectively reflect the thermal stability of the coating in the thermal shock test (medium-low temperature cycle thermal shock) will have more practical significance. 

In this paper, the SSAC of solar energy was prepared on the surface of ultra-pure ferritic stainless steel by chemical coloring for the first time. The effect of coloring potential on the optical properties of the coating was mainly studied. The influence of thermal shock test at 25/300 °C on the micro-structure and thermal stability of the coating was analyzed.

## 2. Materials and Methods

### 2.1. Coloring Process

The sample size is 100 mm × 50 mm × 0.41 mm, and the ultra-pure ferritic stainless steel (SUS430LX, 00Cr18Mo2) is used as the substrate. SUS430LX is a kind of super low carbon nitrogen stainless steel developed in recent decades [26]. The corrosion resistance is improved by adding Ti, Nb and other trace elements. With the following mass composition: 17.4% Cr, 0.32% Si, 0.08% Mn, 0.005% C + N, 0.19% Ti, 0.16% Nb, while the balance was substantially Fe. The aqueous solution of H_2_SO_4_ 81 g/L, Na_2_Cr_2_O_7_·2H_2_O 10.67 g/L was used as the coloring solution and add ZnSO_4_ 1.3 g/L and Cr_2_(SO_4_)_3_ 2.5 g/L as the stabilizer.

**Figure 1:**
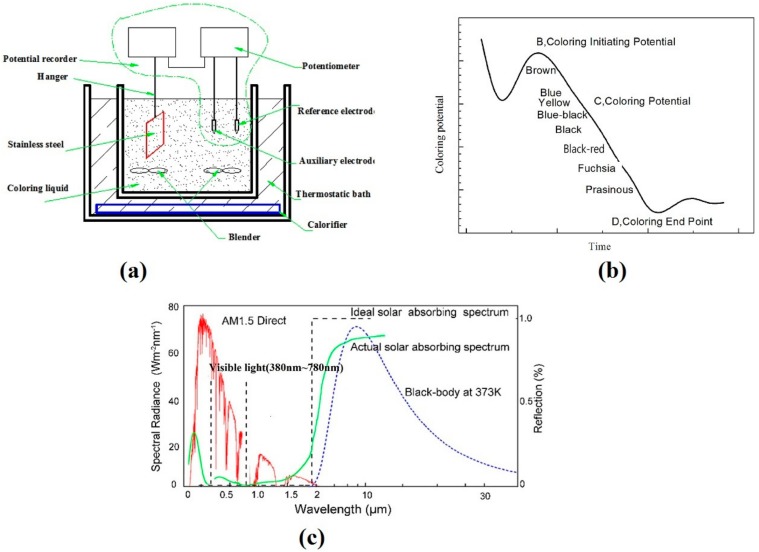
(**a**) Equipment drawing of chemical coloring; (**b**) coloring potential-time curves; (**c**) the spectrum of solar selective.

The first step is to wash the sample in HNO_3_ 8 g/L, DIPSOL st-305 (NH_4_HF_2_ + NaHF_2_ + etc.) 1.3 g/L pickling solution for 60 s to remove surface impurities and activate the surface. The second step was to wash the samples with tap water for 4 times, each time for 30 s. In the third step, the coloring was carried out in the coloring solution at the coloring temperature. Three electrodes (working electrode AISI430, reference electrode platinum electrode) were used to record the coloring potential difference and reaction time of the required color. Schematic diagram is shown in Figure 1a. Finally, four more washes were carried out, each time for 30 s, to effectively remove the coloring liquid and obtain the SSAC.

### 2.2. Metal-Dielectric Composite Coatings 

As shown in Figure 1c, in the case of AM1.5, the solar spectrum of 300–2500 nm accounts for 97.5% of the solar irradiation energy, and the visible band is 380–780 nm [27]. According to the principle of trichromatic color, black and blue black are preferred for the ideal SSAC [20].

The optical properties of the solar absorbing coatings are characterized by two parameters: solar absorptance (α) and thermal emittance (ε), defined as follows:

(2) $$\alpha =\frac{\int_{300}^{2400}I_{s}(\lambda )(1-R(\lambda ))d\lambda }{\int_{300}^{2400}I_{s}(\lambda )d\lambda }$$

(3) $$\varepsilon =\frac{\int_{250}^{2500}I_{b}(\lambda ,T)(1-R(\lambda ))d\lambda }{\int_{250}^{2500}I_{b}(\lambda ,T)d\lambda }$$

where I_s_(λ) is the solar spectral radiation of AM1.5 according to the ISO/9806: 2013, R(λ) is the measured reflectance at a specific wavelength λ, the thermal emittance according to the GB/T 17683, and I_b_(λ,T) is the intensity of spectral radiation of the black body at temperature (T). The solar selectivity (S) of the coatings is calculated using S = α/ε.

The coating obtained on the surface of stainless steel by chemical coloring is a metal-dielectric composite coatings. The surface forms an optical trap structure through corrosion. The trap is filled with oxides. By adjusting the composition, thickness, and particle size of the cermet layer, selective absorption is achieved. At the same time, solar radiation is repeatedly reflected between the translucent dielectric layer and the metal layer will be greatly absorbed, so the absorption can rise.

### 2.3. Coloring Curve

Coloring potential-time curves is shown in Figure 1b. Point B is a point where coloring starts, and a stain starts to form. The potential corresponding to point B is the coloring initiating potential (E_B_), and point C is the coloring point between points B and D. At point C, it is a specific form of color. The potential corresponding to point C is the coloring potential(E_C_), and the point D is the end point of coloring, ΔE is the chemical potential, expressed as E_B_ − E_C_ = ΔE. The color of stainless steel is changed from brown → blue → yellow → blue-black → black → fuchsia → prasinous [21]. Each color corresponds to a different coloring potential when the coloring liquid composition and the substrate composition are constant [22]. In order to obtain the ideal SSAC, first choose the appropriate SSAC color, black or blue black between blue and fuchsia, the control area is more sensitive, so the control of the conditions such as composition and coloring time strict.

### 2.4. Characterization

The coloring potential was recorded by electrochemical workstation (Correst, cs350h, Wuhan, China). the coloring potential-time curve was plotted by scanning rate of 2 mv/s. A UV-Vis-NIR spectrophotometer (Shimadzu, UV-3600, Kyoto, Japan) was used to measure the solar absorptance (α) of the coating at room temperature with the AM 1.5. The wavelength range was set between 300 and 2400 nm, and the total reflectance was corrected by using a BaSO_4_ baseline sample, which was provided by the manufacturer of the spectrophotometer. The thermal emittance (ε) were measured by using a Fourier Transform Infrared (FTIR) spectrometer (Shimadzu, IR-Affinity-1, Kyoto, Japan) at 90 °C with a selected spectral range of 250–2500 nm (4000–400 cm^−1^). The surface composition-analysis of colored film was tested by X-ray photoelectron spectroscopy (Shimadzu, AXIS Supra, Kyoto, Japan). The test conditions for XPS: sample analysis area is 700 × 300 μm^2^, Pass Energy is 100 eV, the excitation source is AlKα(h_ν_ = 1486.6 eV). The basis vacuum of 1.8 × 10^−7^ Pa, corrected by C1s 284.8 eV. The element spectrum through energy is 30 eV respectively.

The thermal shock test used a high-temperature box-type resistance furnace (Website, Sx2-12, shanghai, China) to perform a cyclic thermal shock test on the prepared coating at 25/300 °C. The starting temperature of the experiment was 25 °C, the heating rate was 10 °C/min, the holding temperature was 300 °C, each holding time was 24 h, the cooling rate was 5 °C/min. The Standard “Specification for absorber of flat plate solar collector” (GB/T 26974) stipulates "high temperature durability" "placed in 250 °C air for 200 h" and the PC is not greater than 0.05. The number of cycle test starts for this project is set to 8 (203.2 h in total), 16 (406.4 h), 24 (609.6 h), and 32 times (812.8 h). After the thermal shock test, the coatings were measured for solar absorptance and thermal emittance, respectively, and the PC changes were calculated. The surface homogeneity of colored film was observed via Atomic force microscope (Zeiss, LSM800, Oberkochen, Germany) and Scanning electron microscope (JEOL, JEM-2010, Kyoto, Japan) with Energy dispersive spectrometer (Oxford, X-Max 50, Oxford, United Kingdom). The magnification of SEM was 1 × 10^5^.

## 3. Results and Discussion

### 3.1. Chemical Potential

Without changing the coloring liquid composition, the working temperature was selected to be 118 °C, and the change in the coloring potential was observed through the coloring time. Table 1 shows the potentials corresponding to different colors, and the measurements of solar absorptance and thermal emittance (90 °C) of the corresponding coatings. Through calculation of solar selectivity (*S*), standard sample 2# is the best.

**Figure 2:**
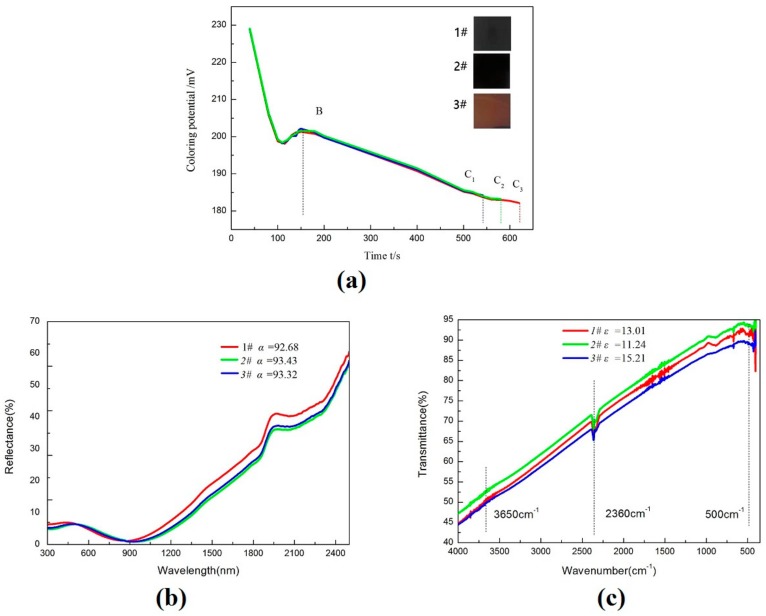
(**a**) Coloring potential-time curves, (**b**) UV-Vis-NIR spectra of the coatings by different samples, (**c**) FTIR spectra of the coatings by different samples.

Figure 2a shows that under the same working conditions, the starting voltage (E_B_) of the same substrate is basically the same. The coloring potential and the color change are consistent during the coloring process. The substrate turns gray in about 150 s and starts to color. The color starts brown → blue → yellow → black-blue → black → Black-red, which is basically consistent with the traditional chemical coloring [23]. Considering the optical performance requirements of SSAC, it is recommended to select the tinting potential of 17.9 mV–19.1 mV. According to this formula, 18.4 mV is preferred, that is, subsequent samples are prepared by 2# process for characterization and a thermal shock test.

The deposited coatings by the three processes all exhibit low reflectance (< 5%) in 3000–1300 nm region (the concentration area of solar radiation energy), and the change from low reflectance to high reflectance abruptly occurred at around 1000 nm, Figure 2b. Note that the selected working temperature is close to ideal. The reflectance in the visible region is close to zero, and it has a high reflectance in the wavelength range of 1300–2500 nm (near infrared and infrared region), which is consistent with the change trend of SSAC coatings prepared by conventional methods [7]. According to the whole reflectance from 300 nm to 2500 nm, the calculated solar absorption is 92.68, 93.43, 93.32. According to the standard of GB/T-6424, the obtained coatings have good absorption performance, which can meet the demand.

Figure 2c shows that all the samples have strong absorption peaks around 500 cm^−1^. The absorption peaks are attributed to the stretching vibrational absorption of spinel chrome iron oxide Fe–O bonds. The two weaker absorption peaks around 2350 cm^−1^ and 3650 cm^−1^ are respectively attributed to the stretching vibration peak and the bending vibration peak of the O–H bond in the adsorbed water contained in the sample. No characteristic absorption peak of Cr^6+^ was found in the infrared spectrum, indicating that Na_2_Cr_2_O_7_·2H_2_O reacted completely with the metal in the substrate.

### 3.2. Analysis of Compositions and Pattern 

**Figure 3:**
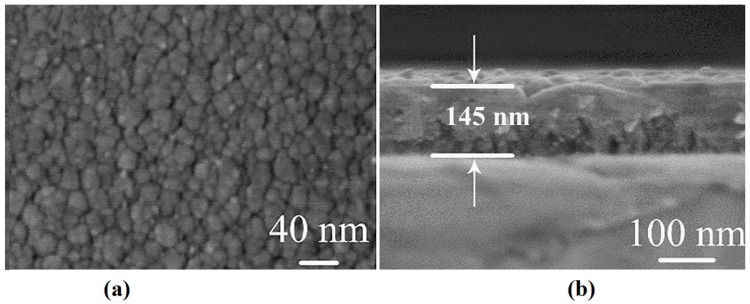
(**a**) SEM surface image, (**b**) SEM cross-section image with the thickness.

Figure 3a shows that the coating surface is dense and uniform. The cross-sectional morphology shown in Figure 3b shows that the coating has a clear interface with the substrate by chemical coloring. The thickness of the coating is about 145 nm. The coating has a layered structure composed of grains and its growth direction is perpendicular to the substrate.

**Figure 4:**
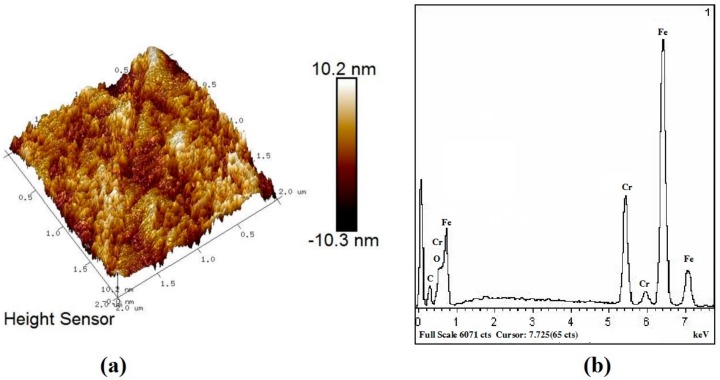
(**a**) 3D AFM image; (**b**) EDS Energy Spectrumof the 2# coatings.

The coating surface obtained by chemical coloration has a nano-scale honeycomb structure with a depth of about 20 nm, as shown in Figure 4a. This structure exhibits optical trapping characteristics and plays a refraction effect. Decreasing the amount of light reflection on the surface increases the energy of light transmission. According to the theory of effective media [28], it has the same properties as adding an antireflection coating on the absorption layer. It is shown that the coatings obtained are mainly oxides of Fe and Cr or an oxidation mixture of the two, and this Chromium-iron oxidation mixture cermet play a role in extensively absorbing the energy of solar radiation, as shown in Figure 4b.

**Figure 5:**
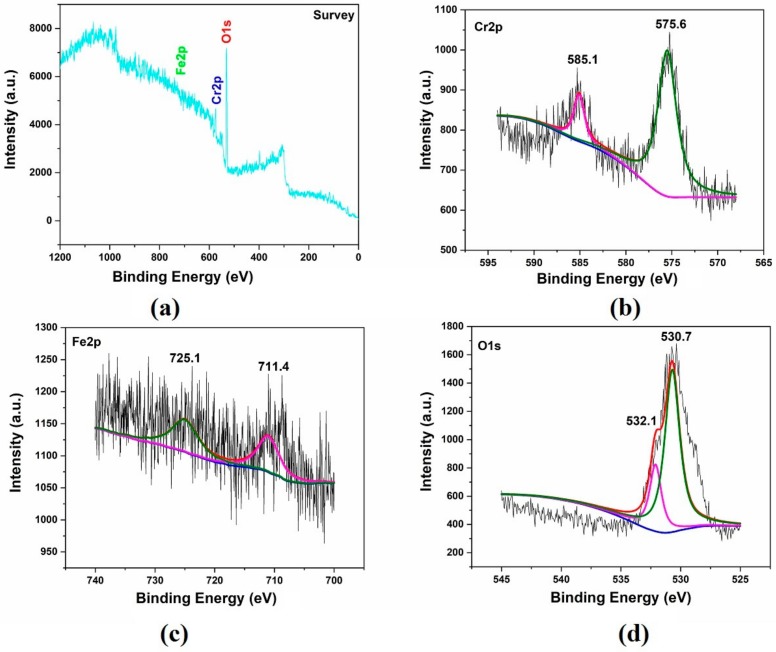
XPS spectra of: (**a**) survey, (**b**) Cr2p, (**c**) Fe2p and (**d**) O1s from the 2# coatings.

As shown in Figure 5, characteristic peaks of coating components were analyzed by XPS. Figure 5a shows the coating Fe2P Cr2P and the core level of O1s XPS through an XPS peak-differentiation-imitating analysis. Cr2P observed component peaks of different intensities, attributed to the oxygen peak of the primary lattice and the formation of hydrates, as shown in Figure 5b. In Figure 5c, Fe2P displays various oxides such as Fe_2_O_3_, Fe_3_O_4_ or FeO. In Figure 5d, the O1s peak has a shoulder at the higher binding region due to the primary lattice oxygen peak and the inherent characteristics of the O1s of spinel or defective oxide components inherent in these composite oxide surfaces.

### 3.3. Thermal Shock Test

**Figure 6:**
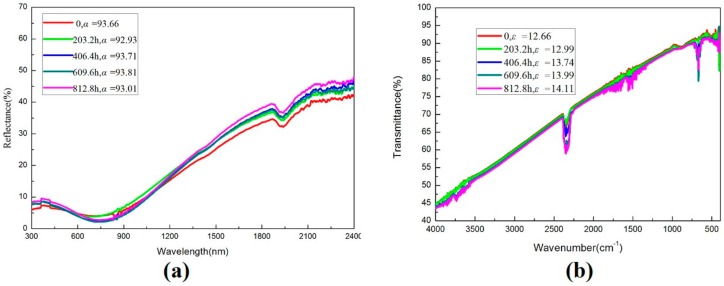
Optical spectrum of: (**a**) UV-Vis-NIR and (**b**) FTIR by thermal shock test.

The SSAC is in the thermal shock of medium and low temperature cycle for a long time, and frequent working temperature changes will lead to the degradation of the coating performance [19]. Thus, the thermal stability of the SSAC of solar energy is very important. Figure 6a,b shows the changes of optical characteristics of the thermal shock test in air at 203.2, 406.4, 609.6 and 812.8 h, indicating that the solar absorptance does not change significantly. The main reason for the increase of PC is the increase of thermal emittance. The corresponding solar absorptance, thermal emittance and PC calculation of the coating are shown in Table 2. In the 32 thermal shock tests up to 25/300 °C (accumulated 812 h), the PC of the coating is 0.01375, which is far less than the standard of 0.05. These results indicate that the coating has good thermal stability in the air.

**Figure 7:**
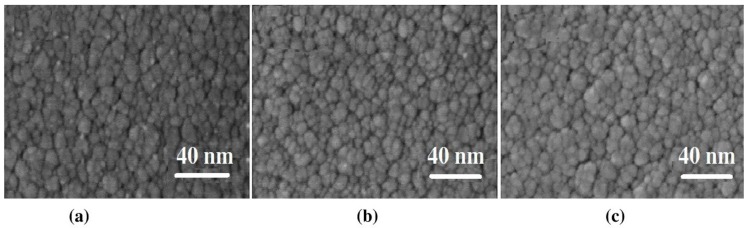
SEM images of by: (**a**) as-deposited, thermal shock test in air for (**b**) 203.2 h, (**c**) 812.8 h.

The surface morphology of the coating was observed by scanning electron microscopy (SEM). As shown in Figure 7, the coating surface features is densely packed nano-sized grains. It is worth noting that after the thermal shock test, the grain boundary becomes blurred and a more compact structure with some particles distributed on the surface is formed. It is speculated that the changes of oxide hydrate in the coating during the frequent thermal shock test lead to the changes of the micro-morphology.

**Figure 8:**
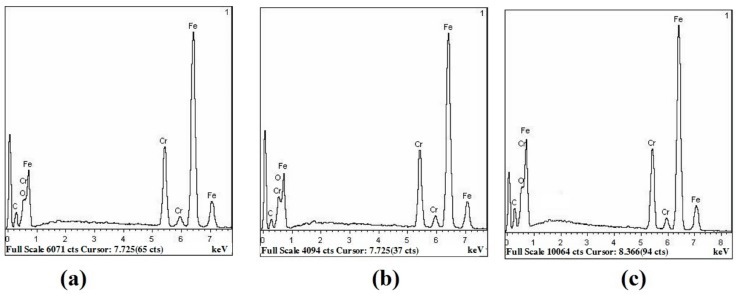
EDS Energy Spectrum of: (**a**) as-deposited, thermal shock test for (**b**) 203.2 h, (**c**) 812.8 h.

The physical properties of chromium-iron oxide have good heat resistance [29]. Figure 8 shows that the main component of the coating obtained by chemical coloring on the surface of the ultra-pure ferritic stainless steel is a chromium iron oxide mixture. Therefore, in the heat resistance test, the EDS spectrum of the coating is relatively stable compared with as-deposited, which is the internal reason for the small change in the solar absorption found in the test of Figure 6a.

## 4. Conclusions

The use of an improved chemical coloring to prepare a solar selective absorption coating on ultra-pure ferritic stainless steel is a feasible solar-thermal coating preparation process. The recommended coloring potential is 17.9 mV–19.1 mV. The SSAC obtained on the surface of ultra-pure ferritic stainless steel (SUS430LX) by chemical coloring, the surface shows an optical trap structure. The coating has excellent optical properties, a solar absorption of 93.66, and a thermal emission of less than 13. After the 32 thermal shock tests at 25/300 °C (accumulated 812.8 h), the coating of Chromium-iron oxidation mixture cermet showed a PC of 0.01375 and an outstanding thermal stability. In the thermal shock test, it was found that the composition of the coating elements was stable and had little effect on the solar absorptance of the coating, mainly because of the slight changes in the surface microstructure and structure.

## Figures and Tables

**Table 1:** The relationship of the potential difference and the color.

Sample	Color Traditional	Coloring Time (s)	Chemical Potential Δ*E*/mV	Absorptance (AM1.5)	Emittance (90 °C)	*S*
1#	Black-blue	560	17.9	0.9268	0.130	7.13
2#	Black	580	18.4	0.9343	0.112	8.34
3#	Black-red	620	19.1	0.9332	0.152	6.14

**Table 2:** Coating performance parameters after thermal shock test.

Test Time (h)	Absorptance (AM1.5)	Emittance (90 °C)	PC
203.2	92.93	12.99	0.00895
406.4	93.71	13.74	0.0049
609.6	93.81	13.99	0.00515
812.8	93.01	14.11	0.01375

## References

[1] Bonhôte P., Eperon Y., Renaud P. (2009). Unglazed coloured solar absorbers on façade: Modelling and performance evaluation. Sol. Energy.

[2] Joly M., Antonetti Y., Python M., Gonzalez M., Gascou T., Scartezzini J.-L., Schüler A. (2013). Novel black selective coating for tubular solar absorbers based on a sol-gel method. Sol. Energy.

[3] Payback S. (2017). Solar heat for industry–Solar Payback. https://www.solarpayback.com/wp-content/uploads/2017/07/Solar-Heat-for-Industry-SolarPayback-April-2017.pdf.

[4] Fernandes J.C.S., Nunes A., Carvalho M.J., Diamantino T.C. (2017). Degradation of selective solar absorber surfaces in solar thermal collectors—An EIS study. Sol. Energy Mater. Sol. Cells.

[5] Khamlich S., McCrindle R., Nuru Z.Y., Cingo N., Maaza M. (2013). Annealing effect on the structural and optical properties of Cr/α-Cr2O3 monodispersed particles based solar absorbers. Appl. Surf. Sci..

[6] Khatibani A.B. (2016). Spray pyrolytically grown NiAlOx cermets for solar thermal selective absorbers spectral properties and thermal stability. Indian Acad. Sci..

[7] Liu J., Sun Z.-Q., Wang H. (2018). Design and characterization of solar absorbing multilayer stack based on Al/ Cr-N-O/SiO2 layers. Sol. Energy Mater. Sol. Cells.

[8] Boubault A., Ho C.K., Hall A., Lambert T.N., Ambrosini A. (2017). Durability of solar absorber coatings and their cost-effectiveness. Sol. Energy Mater. Sol. Cells.

[9] Diamantino T.C., Gonçalves R., Nunes A., Páscoa S., Carvalho M.J. (2017). Durability of different selective solar absorber coatings in environments with different corrosivity. Sol. Energy Mater. Sol. Cells.

[10] Li Z., Zhao J. (2013). Aqueous solution-chemical derived NiAl2O3 solar selective absorbing coatings. 2. Wetting agents and spreading of aqueous solutions on aluminum substrate. Appl. Surf. Sci..

[11] Ma P., Geng Q., Gao X., Yang S., Liu G. (2016). Aqueous chemical solution deposition of spinel Cu1.5Mn1.5O4 single layer films for solar selective absorber. Rsc Adv..

[12] Meng J.-P., Guo R.-R., Li H., Zhao L.-M., Liu X.-P., Li Z. (2018). Microstructure and thermal stability of Cu/Zr0.3Al0.7N/Zr0.2Al0.8N/Al34O60N6 cermet-based solar selective absorbing coatings. Appl. Surf. Sci..

[13] Rodríguez-Palomo A., Céspedes E., Hernández-Pinilla D., Prieto C. (2018). High-temperature air-stable solar selective coating based on MoSi2–Si3N4 composite. Sol. Energy Mater. Sol. Cells.

[14] Köhl M., Heck M., Brunold S., Frei U., Carlsson B., Möller K. (2004). Advanced procedure for the assessment of the lifetime of solar absorber coatings. Sol. Energy Mater. Sol. Cells.

[15] Song P., Wu Y., Wang L., Sun Y., Ning Y., Zhang Y., Dai B., Tomasella E., Bousquet A., Wang C. (2017). The investigation of thermal stability of Al/NbMoN/NbMoON/SiO2 solar selective absorbing coating. Sol. Energy Mater. Sol. Cells.

[16] Soum-Glaude A., Le Gal A., Bichotte M., Escape C., Dubost L. (2017). Optical characterization of TiAlN x /TiAlN y /Al 2 O 3 tandem solar selective absorber coatings. Sol. Energy Mater. Sol. Cells.

[17] Zhang X., Wang X., Zhang X., Li Y., Cheng X. (2018). Effect of multilayered CoO-CoAl2O4 films on improving solar absorptance of Co-WC solar selective absorbing coatings. Vacuum.

[18] Ding D., Wu H., Yu X. (2015). Air-stable NiFeCrOx selective absorber for mid-to-high temperature application. Sol. Energy.

[19] Bayón R., San Vicente G., Morales Á. (2010). Durability tests and up-scaling of selective absorbers based on copper–manganese oxide deposited by dip-coating. Sol. Energy Mater. Sol. Cells.

[20] Sheu H.-H., Lu C.-E., Lee H.-B., Pu N.-W., Wu P.-F., Hsieh S.-H., Ger M.-D. (2016). Electrodeposition of black chromium–cobalt alloy based on trivalent sulfate electrolyte. J. Taiwan Inst. Chem. Eng..

[21] Cheng Z., Xue Y., Ju H. (2018). Chemical coloring on stainless steel by ultrasonic irradiation. Ultrason. Sonochemistry.

[22] Cheng Z., Xue Y., Tang Z., An L., Tian Y. (2008). A one-step process for chemical coloring on stainless steel. Surf. Coat. Technol..

[23] Corredor J., Bergmann C.P., Pereira M., Dick L.F.P. (2014). Coloring ferritic stainless steel by an electrochemical–photochemical process under visible light illumination. Surf. Coat. Technol..

[24] He M., Wang Y., Wang H., Chen R. (2016). A one-step sol–gel route derived Ag–CuO film as a novel solar selective absorber. Sol. Energy Mater. Sol. Cells.

[25] Wang X., Zhang X., Li Q., Min J., Cheng X. (2018). Spectral properties of AlCrNO-based multi-layer solar selective absorbing coating during the initial stage of thermal aging upon exposure to air. Sol. Energy Mater. Sol. Cells.

[26] Kikuti E., Bocchi N., Pastol J.L., Ferreira M.G., Montemor M.F., da Cunha Belo M., Simões A.M. (2007). Composition and structure of coloured oxide films on stainless steel formed by triangular current scan and cathodic hardening treatment. Corros. Sci..

[27] Kim J.-S., Chung W.-S., Kim K., Kim D.Y., Paeng K.-J., Jo S.M., Jang S.-Y. (2010). Performance optimization of polymer solar cells using electrostatically sprayed photoactive layers. Adv. Funct. Mater..

[28] Amri A., Jiang Z.-T., Pryor T., Yin C.-Y., Xie Z., Mondinos N. (2012). Optical and mechanical characterization of novel cobalt-based metal oxide thin films synthesized using sol–gel dip-coating method. Surf. Coat. Technol..

[29] Ma X., Nie X., Zhao J., Han Y., Shrotriya P., Wang Y., Guo J. (2020). Coloring stability analysis of nanosecond pulsed laser induced surface coloring on stainless steel. Opt. Laser Technol..

